# Cherry Antioxidants: From Farm to Table

**DOI:** 10.3390/molecules15106993

**Published:** 2010-10-12

**Authors:** Gianna Ferretti, Tiziana Bacchetti, Alberto Belleggia, Davide Neri

**Affiliations:** 1 Department of Biochemistry, Biology and Genetic – Polytechnic University of Marche, via Ranieri 65, 60100, Ancona, Italy; Email: t.bacchetti@univpm.it (T.B.); 2 SAPROV – Environment and Crop Sciences Department, Polytechnic University of Marche, 60131 Ancona, Italy; Email: d.neri@univpm.it (D.N.); albertbelleg@gmail.com (A.B.)

**Keywords:** sour cherry, sweet cherry, antioxidants, polyphenols, oxidative stress, bioactive compounds, bioavailability, chronic diseases

## Abstract

The dietary consumption of fruits and vegetables is associated with a lower incidence of degenerative diseases such as cardiovascular disease and certain types of cancers. Most recent interest has focused on the bioactive phenolic compounds found in vegetable products. Sweet and sour cherries contain several antioxidants and polyphenols that possess many biological activities, such as antioxidant, anticancer and anti-inflammation properties. The review describes the effect of environment and other factors (such as production, handling and storage) on the nutritional properties of cherries, with particular attention to polyphenol compounds. Moreover the health benefits of cherries and their polyphenols against human diseases such as heart disease, cancers, diabetes are reviewed.

## 1. Introduction

**Figure 1:**
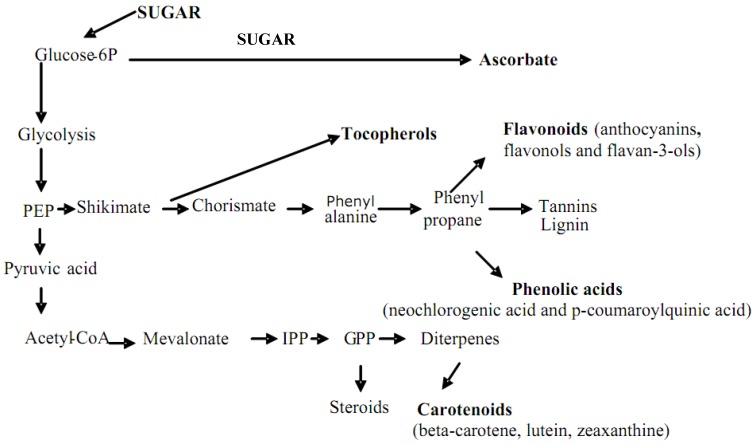
Synthesis of phytonutrients in plant cells. PEP,phosphoenolpyruvate; IPP,isopenthenylpyrophosphate; GPP, geranylpyrophosphate.

Several studies have demonstrated that fruits and vegetables exert a protective effect against the development of human diseases such as cardiovascular disease, diabetes and cancer [1,2,3,4]. It has been hypothesized that the protective role could be due to the various nutrients they contain such as fiber, vitamins and phytonutrients. Phenolics are secondary plant metabolites characterized by having at least one aromatic ring with one or more hydroxyl groups attached. The nature and the distribution of phenolics differ by plant tissue, with many of the phenolics synthesized from carbohydrates via the shikimate and phenyl propanoid pathways (Figure 1). Phenolics range from simple, low molecular weight, single-aromatic ring compounds to the large complex tannins. They can be classified by the number and arrangements of the carbon atoms. These molecules are generally involved in defense against ultraviolet radiation or aggression by plant pathogens. Polyphenols generally occur as glycosylated derivatives in plants, although conjugation with inorganic acid and malonylation are also known. Several studies in animal models and in human subjects have demonstrated that phenols are bioavailable and exert a protective role against oxidative stress and free radical damages [5,6].

Oxidative damage, triggered by free radicals, causes structural and functional alterations of cell macromolecules and is involved in the molecular mechanisms of chronic human diseases [7,8]. Antioxidants have the ability to scavenge or to neutralize free radicals, or are necessary to enable other molecules to perform such a function [8]. Several studies demonstrated that cherries contain several nutrients such as phytonutrients and antioxidants. Aim of this review was to describe the effect of environment and other factors (such as production, handling and storage) on nutritional properties of cherries with particular attention to polyphenol compounds. Moreover the health benefits of cherries and their polyphenols against human diseases such as heart disease, cancers, diabetes will be reviewed. 

## 2. Nutritional Properties of Sweet and Sour Cherries

Cherries (*Prunus* spp.) are the smallest members of the stone fruit family: Rosaceae, genus: *Prunus*; subgenera: Cerasus and Padus. They comprise over a hundred species, classified in pomological terms into two distinct groups based on the type of inflorescence (corymb or racemosa). 

Among the most important species with the corymb inflorescence, belonging to the subgenus Cerasus, *Prunus avium* L., know as "sweet cherry”, and *Prunus cerasus* L., known as "sour cherry or tart cherry” are the most important. The sour cherry varieties are divided in three main groups depending on tree habit and fruit characteristics: Kentish cherries, morellos and marasca.

The main characteristics related to cherry fruit quality are colour, sweetness, sourness, and firmness. Sweetness in the cherry fruit is mainly due to glucose and fructose, while sourness is primarily due to the presence of organic acid (malic acid) [9,10]. The acceptance by consumers seems to be dependent on the ratio between sugar and acid concentrations [11]. Sweet cherries are characterized by a higher content of simple sugar (13 g/100 g) with respect to sour cherries (8 g/100 g). Cherries contain both hydrosoluble (C,B) and liposoluble (A, E and K) vitamins and some carotenoids, in particular *beta*-carotene, and to a lower extent lutein and zeaxantine (Table 1). As summarized in Table 1, sour cherries are characterized by an higher content of vitamin A and beta-carotene. Cherries contain also minerals such as calcium (14 mg/100 g), magnesium (10 mg/100 g), phosphorous (20 mg/100 g) and potassium (200 mg/100 g). In two tart cherry cultivars Burkhardt *et al.* demonstrated the presence of melatonin (13.46 ± 1.10 ng/g and 2.06 ± 0.17 ng/g in Balaton and Montmorency, respectively) [12]. As shown in Table 1, sour cherries contain a higher level of total phenolics than sweet cherries.

**Table 1:** Phytonutrients in sweet and sour cherries (in 100 g).

	Sweet cherries	Sour cherries
**Vitamin C**	7 mg	10 mg
**Niacin**	0.2 mg	0.4 mg
**Pantothenic Acid**	0.2mg	0.1mg
**Vitamin A**	64 IU	1283 IU
**Vitamin E (Alpha Tocopherol)**	0.1mg	0.1mg
**Vitamin K**	2 .1μg	2.1 μg
**Beta Carotene**	38 μg	770 μg
**Lutein+Zeaxanthin**	85 μg	85 μg
**Total Phenols**	109.8 mg	228.9 mg

### 2.1. Polyphenols in cherries

In cherry, as in other red fruits, the ripening process is related to a change from the initial green color to red, with accumulation of polyphenolic compounds, anthocyanins and degradation of chlorophyll. Phenolic compounds are concentrated in the skin and contribute to sensory and organoleptic qualities of fruits, such as taste and astringency. Furthermore, it has been demonstrated that they are bioactive compounds. The phenols contained in sour and sweet cherries have been characterized [13]. Cyanidin 3-glucoside, cyanidin 3-rutinoside, cyanidin 3-sophoroside, pelargonidin 3-glucoside, pelargonidin 3-rutinoside, 3-glucoside, and peonidin 3-rutinoside have been identified in sweet and sour cherries [14,15,16,17,18].

Among phenolic acids, hydroxycinnamates (neochlorogenic acid and p-coumaroylquinic acid) have been quantified either in sweet and sour cherries. Flavonols and flavan-3-ols such as catechin, epicatechin, quercetin 3-glucoside, quercetin 3-rutinoside, and kaempferol 3-rutinoside were also found in sweet and sour cherries [15,16]. The higher levels of total phenolics in sour cherries have been attributed to higher concentrations of anthocyanins and hydroxycinnamic acids [19] (Table 2).

As summarized in Table 2, different cultivars of sweet and sour cherries show a high variability of phenol compound levels. Total anthocyanins of sweet cherries are between 30 (cv. Black Gold) and 79(cv. Cristalina) mg cyanidin-3-glucoside equivalents (CGE)/100 g, whereas total anthocyanins of sour cherries were between 45(cv. Balaton) and 109 (cv. Sumadinka) mg CGE/100g [13,19].

**Table 2:** Total anthocyanins and total phenols in different cultivars of sweet or sour cherries ^a^ from Serrano *et al* (2009); ^b^ from Kim DO *et al.* (2005); GAE, gallic acid equivalent; CGE, cyanidin-3-glucoside equivalents.

Cultivar	Total phenols (mg GAE/100g fresh cherries )	Total anthocyanins (CGE/100g fresh cherries)
**sweet Brooks^a^**	60 ± 13	10 ± 2
**sweet Newstar^a^**	75 ± 14	20 ± 5
**sweet Black Gold^b^**	92 ± 12	30 ± 9
**sweet Hedelfingen^b^**	96 ± 20	40 ± 7
**sweet Regina^b^**	104 ± 6	41 ± 2
**sweet Hartland^b^**	147 ± 19	76 ± 12
**sweet Cristalina^a^**	155±20	79 ± 5
**sour Balaton^b^**	254.1 ± 6.0	45 ± 2.3
**sour Danube^b^**	162 ± 1	65 ± 3
**sour Schattenmorelle^b^**	295 ± 34	72 ± 6
**sour Sumadinka^b^**	312 ± 8	109 ± 6

### 2.2. Effect of pre-harvest and post harvest conditions on cherry nutritional properties

Genetic and environmental factors modulate the composition and concentration of macronutrients, micronutrient and phytonutrients in plant foods [16,20,21].

#### 2.2.1. Effect of pre-harvest factors

Pre-harvest factors and temperature, light intensity, fruit crops maturity may affect the content and stability of phytochemicals and the nutritional value in cherries. Light intensity increases levels of ascorbic acid and different growing temperatures (day/night) also affect total phenolic content. High temperature growing conditions (25/30 °C) significantly enhance anthocyanin and total phenolic content [22]. Also soil types, composts, mulching and fertilization influence the water and nutrient supply to the plant and can affect the nutritional composition of the harvested fruit. 

Actually, in cool growing areas interest is increasing in growing cherries under plasticulture systems to prevent cherry cracking and to favour harvest operations. This cultivation system can influence canopy and soil temperature, quantity and quality of transmitted, reflected or absorbed light, with a possible difference in fruit growth, development, quality or carbohydrate metabolism. For example, in strawberry black plastic mulching consistently resulted in higher flavonoids, anthocyanin and total phenolic content compared to the traditional matted row culture system [22]. 

Actually in all cherry growing regions different rootstock are used for many reasons related to climate, environment, field and orchard training. ‘PiKu 1’ and ‘Weiroot 13’ rootstocks gave fruits of Lapins cultivar with significantly higher phenolic acid and flavonol content [23]. Higher amounts of phenols in sweet cherry fruits grown on heterogenic graft combinations might be attributed to the adjustment of the metabolism to genetically different between rootstocks and varieties, which causes higher amounts of metabolic stress [24]. Trees of different vigor can produce sweet cherry fruits with different phenolic acid and flavonol content. Wild growing sweet cherry genotypes have different nutritional properties and are relatively higher in blackish skin colored fruits than in light skin colored ones [25]. 

#### 2.2.2. Post-harvest factors

Post harvest factors such as transport and storage can also influence phytochemical composition of food crops. In some cases, an increase in phenolic content has been observed in the days following fruit purchase and they are generally stable during storage [26]. For all cultivars and maturity stages, during postharvest storage the ripening process advances, acidity decreases, and color intensity increases as well as anthocyanins. During cold storage and subsequent shelf-life, a general increase (over 40–60% on average) in phenolic compound is observed [27,28]. 

Modern technology could indirectly increase the nutritional value of fruits through the delay of the softening process so that fruits could be harvested at a later, more mature stage, when more of the phyto-compounds have been bio-synthesized [22].

#### 2.2.3. Cherries processed products

Sweet cherries (*Prunus avium* L.) are mainly consumed as fresh fruit. Processed cherries are available all year-round: dry (with or without sugar), frozen, as juice or concentrate, in IQF (individually quick frozen) powder, and canned [15]. During canning, approximately half the anthocyanins and phenolics leach from the fruits into the syrup with little total loss. Spent cherry brine contains substantial anthocyanins and phenolics [15]. Recently we demonstrated that the levels of total phenolic compounds are higher in fully ripen sour cherries when compared to early ripen fruits (unpublished results). Similar results have been reported also in different cultivars of sweet cherries [28]. 

Frozen sour cherries derived from Balaton and Montmorency have the highest levels for both anthocyanins and total phenolics and products processed with sugar (15% of total fresh weight) show lower concentrations for both anthocyanins and phenolic than dry products [29]. Heat processing does not result in a loss of total anthocyanins and total phenolics when the values for syrup and cherries were combined [15].

## 3. Cherry Phenols Bioavailability and Physiological Roles

Bioavailability of dietary polyphenols and tissue concentrations have been previously investigated. It has been demonstrated that bioavailability varies widely among polyphenols and, for some of compounds, among dietary sources, depending on the forms they contain [30,31]. Furthermore, fruit maturity state could also modulate bioavailability as demonstrated by Fazzari *et al*. [32], using sweet cherries. Food polyphenols are mainly found in glycosylated forms, and glycosylation influences absorption [31]. In fact the aglycones can be absorbed from the small intestine, while polyphenols present in food in the form of esters, glycosides, or polymers must be hydrolyzed by intestinal enzymes or by the colonic microflora before they can be absorbed. After absorption, phenols are conjugated in the small intestine and later in the liver. This process is highly efficient and mainly includes methylation, sulfation and glucuronidation. Circulating polyphenols are conjugated derivatives that are transported bound to albumin to peripheral tissues where they can exert physiological roles. Finally, they are eliminated chiefly in urine and bile [31]. 

Polyphenols have been quantified in several tissues reaching a concentration ranging from 30 to 3,000 ng aglycone equivalents/g tissue, in relation to the dietary intake [31,33]. The plasma kinetics differ among polyphenol classes, with higher concentrations reached after about 1.5 h or 5.5 h, depending on the site of intestinal absorption [34]. Plasma concentration reached after phenol consumption varies highly according to the nature of phenols and food source with values ranging 0.5–1.6 μM [31]. Human genetic differences could lead to differences in absorption and metabolic clearance between individuals, in addition to differences in intestinal microorganism populations [35]. As far as concerns tannins, they are very poorly absorbed and may exert only local activity in the gastrointestinal tract [31]. 

### 3.1. Bioactivity of phenolic compounds from sour and sweet cherry

**Figure 2:**
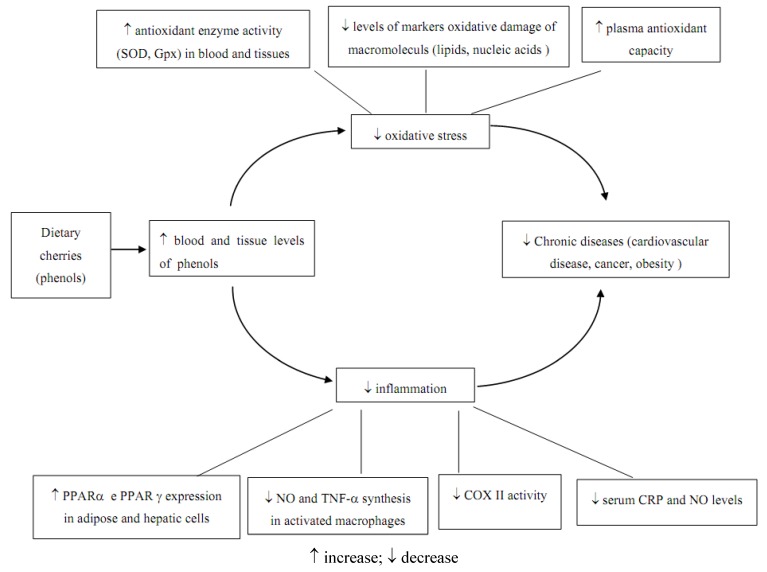
Protective effects exerted by cherry phenols and implication in chronic diseases.

Cherries contain several phenolic compounds whose biological activities have been recently investigated in different experimental models. Antioxidant, anti-inflammatory and anticancer properties have been demonstrated and the molecular mechanisms are summarized in Figure 2.

#### 3.1.1. Antioxidant activity

The antioxidant capacity of extracts from cherries has been widely investigated using different methodological approaches. Using an ORAC (oxygen radical absorbance capacity) assay, it has been reported that the antioxidant capacity ranges from 1,145 to 1,916 μmol Trolox equivalents (TE)/100 g in sour cherries (Amarena Mattarello, Visciola Ninno and Visciola Sannicandro) [36]. It has to be stressed that the values found in sour cherry fruits are comparable to those found in some berry fruits, for example, strawberry, and are higher than in apple and kiwi fruit [37]. 

The antioxidant activity of cherry extracts has been studied also in different biological samples and Tsuda *et al*. [38,39] demonstrated, among the anthocyanins tested, cyanidin 3-glucoside, showed stronger antioxidant activity. Using liposomes as model membranes, it has been reported that anthocyanins, cyanidin and hydroxycinnamates isolated from tart or sweet cherries are more active compared to those of other berries (such as blackberries, red raspberries, blueberries or strawberries) [40]. Furthermore, extracts of sweet cherries are able to inhibit copper triggered low density lipoproteins (LDL) oxidation, an atherogenic modification of this lipoprotein class [40]. The antioxidant activity of cherry phenolics was confirmed using neuronal PC 12 cells exposed to oxidative stress [41]. 

Cherries show antioxidant effects also *in vivo* (Figure 2). An increased activity of the antioxidant enzymes SOD (liver, blood) and Gpx (liver) and a decrease of lipid peroxidation were observed in animal model fed with sour cherry juices obtained from an autochthonous cultivar (*Prunus cerasus* cv. Maraska) [42]. Studies in human subjects, reported that the consumption of 280 g of cherries (about 45 sweet Bing cherries) increased plasma lipophilic antioxidant capacity [43,44]. Moreover, in a double-blind, placebo-controlled crossover design, tart cherry juice intake (240 mL twice daily for 14 d) was associated with a decrease of F(2)-isoprostane levels, a marker of oxidative damage, in response to forearm ischemia-reperfusion. In the same subjects a decrease of urinary excretion of markers of oxidized nucleic acids (8-hydroxy-2'-deoxyguanosine, 8-hydroxyguanosine) was demonstrated [45]. In addition to anthocyanins also phenolic acids could contribute to antioxidant activity of cherries [40].

#### 3.1.2. Anti inflammatory effect

Several studies have demonstrated that cherry intake inhibits inflammatory pathways. Kelley *et al* [46] have shown a decrease of blood levels of C-reactive protein (CRP) and nitric oxide (NO) in healthy subjects after intake of sweet cherries (280 g/die) for 28 days [46]. These results are in agreement with other studies [44]. Elevated levels of serum CRP is one of the most important indicators of inflammation and it is a significant risk factor for cardiovascular disease (CVD) [47]*.* The decrease in plasma CRP levels after cherry intake suggests a reduction in inflammation that may affect the risk for cardiovascular diseases. Also the increased production of NO and their reaction product peroxynitrite (ONOO-) contribute to oxidative stress, tissue injury and an increase of their plasma concentrations are implicated in a variety of rheumatic diseases, including systemic lupus erythematosus, rheumatoid arthritis and osteoarthritis [48,49]. It has been suggested that the decrease in plasma NO level probably results from inhibition of the enzyme nitric oxide synthase–inducible (iNOS) activity [46]. Studies *in vitro* have demonstrated that anthocyanins are able to inhibit nitric oxide (NO) production and other pro-inflammatory factors such tumor necrosis factor-alpha (TNF-α) in activated macrophages [50,51]. Therefore, it has been suggested that the anti-inflammatory properties of cherries could be mainly related to anthocyanins.

Studies in *vitro* and *in vivo* have demonstrated that anti-inflammatory properties of cherry phenols could also due to inhibition of the activity of the cyclooxygenase II (COX II) [42,52,53]. COXs are pro-inflammatory enzymes that play an important role in processes such as inflammation, carcinogenesis, apoptosis, cell proliferation and angiogenesis. It has to be stressed that the cyclooxygenase inhibitory activities of cherry anthocyanins are comparable to those of ibuprofen and naproxen at 10 μM concentrations [52,53]. 

The inhibitory effect of inflammatory pathways has been hypothesized to be the main molecular mechanism to explain the relationship between the consumption of cherries or cherry products and the alleviation of arthritic pain and gout [54,55,56,57,58]. 

Obesity is associated with inflammation. Using model animals (Zucker fatty rat model of obesity and metabolic syndrome and Dahl Salt-Sensitive rat) it has been demonstrated that tart cherry-enriched diets reduce plasma and tissue inflammation. In fact it has been reported that cherry intake for 90 days significantly reduced the levels of proinflammatory molecules in plasma (interleukin-6, TNF-α) and the activity nuclear factor kappa and increases peroxisome proliferator-activated receptors (PPARs) in abdominal fat and in hepatic tissues. These modifications were associated with a decrease of levels of serum glucose, triglycerides and cholesterol [59,60].

### 3.2. Anticancer properties

Studies *in vitro* using several cell culture systems including colon, endothelial, liver, breast and leukemic cells and keratinocytes have demonstrated that anthocyanins exhibit multiple anti-carcinogenic effects [61]. Potential cancer chemopreventive activities of anthocyanins revealed from *in vitro* studies include also their ability to stimulate the expression of Phase II detoxification enzymes (glutathione reductase, glutathione peroxidase and NAD(P)H: quinone reductase) and to inhibit mutagenesis by environmental toxins and carcinogens. Moreover, anthocyanins are able to induce cellular apoptosis and differentiation and to reduce cellular proliferation by modulating different signal transduction pathways (PI3K/Akt, ERK, JNK, and MAPK pathways) [61].

As far as concerns cherry anthocyanins, studies *in vitro* have demonstrated that they are able to reduce proliferation of human colon cancer cells in culture [62]. Moreover, using APC(Min) mice, a model for human colon cancer, it has shown that tart cherry extract intake (375–3,000 mg/kg diet) inhibit tumor development [62,63].

## 4. Conclusions

Several studies have shown that the prevention of cancer, cardiovascular disease and obesity are beneficial health effects attributable to phytocompounds found in fruits and vegetables. The significant decrease of markers of inflammation and oxidative stress afforded by cherries and derivates may have implications for the management of clinical pathologies associated with high levels of inflammation and oxidative stress and suggests that their consumption may have the potential to reduce cardiovascular or chronic diseases in humans (Figure 2). Cherries contain higher amounts of phytocompounds, in particular of phenols when compared to several other fruits, but with high variability among different cultivars, the highest phenols contents being in the sour cherries. Not only genetics, but also environmental factors and cultivation techniques may modulate the composition and concentration of macronutrients, micronutrient and phytonutrients in the cherries. In particular pre-harvest day and night temperature, light intensity, and fruits maturity may affect the content and stability of phytochemicals. Cherry orchard management and fertilization are also important and may strongly affect tree vigor and finally fruit phenolic acid and flavonol content. Modern technology could indirectly increase the nutritional value of fruits, protecting the fruits from the cracking and delaying the softening process so that fruits could be harvested at a later, more mature stage, with higher phyto-compounds content. The type of consumption is also important: sweet cherries are mainly consumed as fresh fruit and tart cherries are generally processed. It is worth noting that modern processing techniques do not result in a loss of total anthocyanins and total phenolics, thus allowing having an all-around-year consumption of healthy delicious cherry fruits.
